# Synthesis of High-Water-Resistance Lignin-Phenol Resin Adhesive with Furfural as a Crosslinking Agent

**DOI:** 10.3390/polym12122805

**Published:** 2020-11-27

**Authors:** Yufei Zhang, Ning Li, Zhikang Chen, Chen Ding, Qi Zheng, Jindi Xu, Qiulu Meng

**Affiliations:** School of Resources, Environment and Materials, Guangxi University, No. 100 Daxue East Road, Nanning 530004, China; zhangyufeifei66@163.com (Y.Z.); czk1119@163.com (Z.C.); dingchen1818@126.com (C.D.); zhengqist@163.com (Q.Z.); xjd1915301051@163.com (J.X.); MengLib06@163.com (Q.M.)

**Keywords:** lignin, furfural, phenolic resin

## Abstract

In this study, furfural was used as a crosslinking agent to enhance the water resistance of lignin-phenol-formaldehyde (LPF) resin. The effect of the furfural content on the physicochemical properties of the adhesives was explored, and the possible synthesis mechanism of the furfural-modified lignin-phenol-formaldehyde (LPFF) resin adhesives was investigated. Compared with the LPF adhesive, the LPFF adhesive with 15% furfural content and 50% lignin substituent exhibited outstanding properties in all considered aspects; it had a high wet shear strength (1.30 MPa), moderate solid content (54.51%), and low viscosity (128 mPa∙s), which were 38.0% higher, 3.6% higher, and 37.5% lower than those of the LPF adhesive. Analyses via nuclear magnetic resonance and Fourier transform infrared (FTIR) spectroscopy confirmed that the furfural content improved water resistance of the lignin-based adhesive; this improvement was due to the formation of new chemical bonds between furfural and lignin to construct a dense crosslinked network structure. In addition, the decrease in viscosity and the increase in solid content enabled the adhesive to better penetrate into the wood porous structure, showing stronger adhesion. Therefore, the LPFF adhesive has superior water resistance, high strength, and good thermal stability; thus, it has a great potential for industrial applications.

## 1. Introduction

Phenolic formaldehyde (PF) resin is currently the most extensively used macromolecular material, which can be prepared by condensation of phenols and aldehydes under the catalysis of acid or alkali. PF resin has always now widely used for national defense and military industry, construction, automobile, aerospace, composite materials, ceramic fabrication including sand casting (3D printed sand cores), adhesives, and other fields because of its heat resistance, anti-flaming, wear resistance, and excellent mechanical properties. Among these applications, PF resin adhesive has always played an important role in the wood processing industry because of its excellent temperature stability, resistance to moisture, and high mechanical strength [1,2,3]. Nevertheless, some vexing drawbacks, including high material cost, high toxicity and easy glue penetration, has severely restricted its application. Biomass resources are natural polymer materials with wide sources, low prices, non-toxic and renewable resources. Therefore, the development and utilization of biomass resources have become a hotspot in social research.

Lignin is a renewable aromatic resource in nature and maybe replace phenol to prepare lignin-phenol-formaldehyde (LPF) resin adhesive [4,5,6,7,8], due to the large number of phenolic hydroxyl and aliphatic hydroxyl groups in its structure. This method can effectively reduce the production cost and free formaldehyde content in PF resin. However, due to the high relative molecular weight and aromatic ring hindrance of lignin, the reaction activity is insufficient, which even hinders the normal condensation of phenol and formaldehyde, resulting in the decrease of water resistance and the increase of viscosity of PF resin [9,10]. Therefore, a variety of studies have been carried out to improve the activity of lignin, but studies to effectively resolve the problem of water resistance decline when lignin replaces phenol with a high proportion were relatively rare. Several strategies have also been directed to improve the water resistance of LPF resin adhesive. On the one hand, scholars have tried to develop multiple methods to realize the efficient utilization of lignin, such as sulfonation, phenolation, demethylation, hydrolysis, hydrogenation, and oxidative cracking [11,12,13,14,15]. On the other hand, reagents were introduced into the adhesive. Dong et al. used lignin to combine with Al^3+^, the hydrophilic groups (–OH,–COO–) are combined with the wood fibers by Al^3+^ in the surface sizing process, the hydrophobic groups (phenylpropane) can stretch orient to face outwards and form a continuous film layer, which tends to be water repellent [16]. These methods increase the cost and the process is complex. Therefore, it is attractive, but challenging, to develop an effective method to increase the water resistance of LPF resin. For decades, LPF resin has generally been considered to be a thermosetting polymer, its physical and mechanical properties and water resistance are closely related to the degree of molecular crosslinking and condensation. Studies have shown that promoting the polymer to form a dense network structure can inhibit the excessive expansion of the adhesive in the process of high-temperature compression and water absorption [17]. Obviously, promoting the formation of the crosslinking network in the condensation seems to be a feasible approach to solving the water resistance issue of LPF resin. At present, resorcinol, melamine, glyoxal, and imidazole have been widely and commercially used to improve the mechanical strength of LPF resin by increasing the molecular interaction. Wang et al. used the nitrogen of the imidazole group was able to form hydrogen bonding interactions with the OH groups in lignin [18]. Nonetheless, the production of synthetic polymers has increased environmental pressure due to the lack of sustainability. Consequently, it is imperative and desirable to improve the water resistance of LPF resin through an effective and renewable approach.

Furfural is obtained from the processing residues of corn, wheat, sugarcane, and other agricultural products. Due to its unsaturated double bond, oxygen ether bond, diene, and other functional groups, furfural has high chemical activity and good heat and water resistances. It has been widely used in the materials and food industries. According to reports, furfural has proved to be a potential crosslinking agent. Adding an appropriate amount of furfural to a synthetic resin system can facilitate the resin polycondensation reaction, thereby improving the physical and mechanical properties [19,20]. At present, formaldehyde-free melamine resin, phenol-furfural resin, and resorcinol-furfural resin have been successfully prepared with furfural as a condensation agent [21,22,23,24]. The idea of using furfural to improve the degree of crosslinking of LPF resin adhesive was inspired by these studies. Meanwhile, the interaction between furfural and lignin, and the influence of furfural on the physicochemical properties of LPF adhesive remain to be explored. Therefore, the current study aims to develop a lignin-based adhesive with good water resistance and a more stable multi-dimensional network structure through a simple process by introducing furfural as a crosslinking agent. In addition, the use of furfural promotes the rational utilization of agricultural waste.

In this study, furfural was used as a crosslinking agent to prepare a high-strength and high-water-resistance furfural-modified lignin-phenolic-formaldehyde (LPFF) resin adhesive. The wet shear strength of the specimens was examined to evaluate the water resistance. The effects of adding different proportions of furfural on the basic properties of LPF adhesives were explored. The morphological changes in the wood bonding joints after the shear strength test of different adhesives were systematically analyzed via scanning electron microscopy (SEM). Moreover, the possible crosslinking mechanism of furfural was investigated through Fourier-transform infrared (FTIR) and nuclear magnetic resonance (NMR) spectroscopy techniques. Furthermore, the thermal stability of the resin was evaluated via thermogravimetric analysis (TGA) and derivative thermogravimetry (DTG).

## 2. Materials and Methods 

### 2.1. Materials

The KL was obtained from Hunan Taigreen Paper Group Hongjiang Paper Co., Ltd (Huaihua, China). Lignin was extracted by filtering and removing some alkali and sugar through membrane treatment, and its pH was 6.7. The dried lignin was ground with a ball mill to a particle size of less than 124 μm and stored. Phenolic resin (PF; shear strength = 1.27 MPa, solid content = 39.40%, viscosity = 65 mPa·s, free phenol = 0.68%, free aldehyde = 0.13%, pH = 11.2) was purchased from Guangxi Guigang Lieran Chemical Co., Ltd (Guigang, China). Phenol, formaldehyde, furfural, hydroxylamine hydrochloride, and sodium hydroxide were purchased from Chengdu Kelong Chemical Co., Ltd. (Chengdu, China), and directly used in experiments. The chemicals used were of analytical grade. The eucalyptus veneers used for manufacturing the plywood were provided by a Qiaosheng wood industry company in Guangxi province, Nanning, China. 

### 2.2. Synthesis of LPF Adhesive and LPFF Adhesive

**Figure 1:**
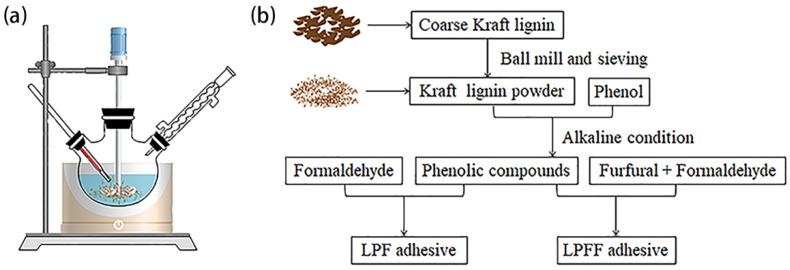
Experiment schematic: (**a**) Equipment diagram; (**b**) experimental flowchart.

The experimental setup is shown in Figure 1a. The KL, 25 g phenol, and sodium hydroxide (40%, *w*/*w*) were loaded into a 500 mL four-necked flask equipped with a reflux condenser, thermometer, and mechanical stirrer. Under stirring, the flask was heated on a water bath preheated to 80 °C for 120 min. Then 70% of a solution of formaldehyde and furfural was added under alkaline conditions and 90 °C, and the system was stirred continuously for 50 min. Subsequently, the remaining 30% of the formaldehyde and furfural solution was added to the reaction system. After 40 min of condensation polymerization the system was immediately cooled to 70 °C and slowly brought to room temperature to terminate the reaction. The LPFF adhesive was then obtained. The experimental process is illustrated in Figure 1b. 

In the experiment, the molar ratio of phenol to formaldehyde was kept at 1:1.6, and sulfate lignin replaced 50% of phenol. Furfural was added at a mass ratio of 0%, 5%, 10%, 15%, 20%, 25%, and 30% (m furfural/[m lignin + m phenol]). For better representation, the corresponding adhesives are denoted as LPF, LPFF5, LPFF10, LPFF15, LPFF20, LPFF25, and LPFF30, respectively.

### 2.3. Preparation of Three-Layer Plywood and Water-Resistance Test

Three single layers of plywood were prepared using eucalyptus veneer with a size of 325 × 325 × 1.8 mm^3^. The spread rate of adhesive per one glue line was 150 g/m^2^. The plywood was then pre-pressed at room temperature for 15 min and hot-pressed for 6 min under 1.2 MPa pressure at 160 °C. After the plywood was kept at room temperature for 24 h, it was cut into 100 × 25 mm samples. Six samples of each adhesive were randomly selected, and the shear strength was determined according to the requirements of Class I boards in the Chinese National Standard (GB/T 17657-2013). The specimen was boiled in water for 4 h, after which it was dried at 63 °C for 20 h. It was boiled again in water for another 4 h, subsequently cooled for 10 min, and then used for tests.

### 2.4. Evaluation of Resin Properties

Various properties of the synthesized LPF and LPFF adhesives such as solid content, viscosity, and pH were determined according to the matching Chinese National Standard (GB/T 14074-2017). The non-volatile (solid) contents were determined through evaporation of the resin samples at 120 °C for 120 min before weighting and calculating. Viscosity of wood adhesives was measured using a rotational viscometer at 23 °C (Shanghai Jinghui Instrument equipment Co. Ltd, Shanghai, China). The recorded viscosity values as average from triplicate measurement were in mPa·s unit. The pH was measured with an electronic pH meter from Shanghai Puchun Measuring Instruments Co., Ltd., model PHS-3C (Shanghai, China). 

### 2.5. Free Aldehyde Determination by Titration

The measurement methodology followed as described in China National Standard (GB/T 14074-2017). The free aldehyde level is determined with hydroxylamine hydrochloride standard method. According to the principle that free aldehyde is easy to oximate with hydroxylamine hydrochloride, the HCl formed by the reaction is titrated with NaOH, and the content of free aldehyde is calculated according to the consumption of NaOH solution. The oximation reaction is:

CH_2_O + H_2_N-OH·HCl → H_2_CN-OH + HCl + H_2_O

Specifically, 5 g sample was dissolved in beaker with 50 mL CH_3_OH, and the pH value of solution was adjusted to 3.5 with HCl. Then drop 25 mL of hydroxylamine hydrochloride solution was added and stirred for 10 min at 25 °C. The pH of tested solution was rapidly titrated to 3.5 by 0.05 mol/L NaOH solution. At the same time, a blank test is performed. The free aldehyde mass fraction is calculated according to the Equation (1):

(1) $$w=\frac{\left(v_{1}-v_{0}\right)\times 0.03003\times c}{m}$$

where w is the content of free formaldehyde. *v*_1_ and *v*_0_ are the volume of NaOH solution consumed to titrate the sample and the corresponding blank sample, respectively, and 0.03003 means the mass of formaldehyde was equivalent to 1.0 mL NaOH (0.05 mol/L). c represents the actual concentration of NaOH solution, and m is the quality of the tested sample.

### 2.6. Free Phenol Determination by Titration

The measurement methodology followed the China National Standards (GB/T 14074-2017). The unreacted phenol in the resin is distilled out by steam distillation, and the free phenol content is determined by bromometry. Specifically, a 2 g sample was dissolved in 100 mL distilled water, and 500 mL distillate was obtained in 50 min using a distillation device. The 50 mL distillate, 25 mL KBrO_3_-KBr, and 5 mL HCl were pipetted into an iodine bottle and treated in the dark for 15 min. Then 1.8 g KI was added and let stand for 10 min. Finally, starch indicator was added when the solution was titrated with Na_2_S_2_O_3_ standard solution to pale yellow, and continued to titrate until the blue color disappears. At the same time, a blank test is carried out. The free phenol mass fraction is calculated according to the Equation (2):

(2) $$p=\frac{\left(v_{1}-v_{2}\right)\times c\times 0.01568\times 1000}{m\times 50}$$

where *p* is the content of free phenol. *v*_1_ and *v*_2_ are the volume of Na_2_S_2_O_3_ consumed by the blank sample and the corresponding sample to be tested, respectively. c is the concentration of Na_2_S_2_O_3_ solution, 0.01568 means the molar mass of phenol equivalent to 1.0 mL Na_2_S_2_O_3_ (0.1 mol/L). m represents the quality of the test sample.

### 2.7. Characteristics

The FTIR data of the adhesive samples were recorded from 4000 to 400 cm^−1^ with a Thermo Scientific Nicolet iS50 spectrophotometer (Waltham, MA, USA). For each adhesive sample, 32 scans were performed at room temperature, with a resolution of 4 cm^−1^. For all dried adhesives, the spectra were collected in the solid state, using KBr pellets. The chemical structure of the LPFF adhesive was characterized via ^1^H-NMR and ^13^C-NMR. Approximately 40 mg of the freeze-dried sample was dissolved in deuterated dimethyl sulfoxide (DMSO-d6). The analysis was conducted at room temperature, using a Bruker Avance 600 MHz (Bruker, Karlsruhe, Germany). The thermal stability of adhesive samples was tested via TGA, using a Shimadzu DTG-60(H) instrument (Shimadzu, Kyoto, Japan). Samples (8 mg in alumina pan) were heated from 50 °C to 800 °C at a rate of 10 °C/min. The surface characteristics of bonded joints were observed using a scanning electron microscope (Hitachi, S-3400N, Tokyo, Japan) at an operating voltage of 5000 V. Bonded joints that were subjected to the shear strength test were selected for drying treatment, and they were observed after gold plating with an anion sputter-coating instrument.

## 3. Results and Discussion

### 3.1. Water Resistance of Wood-Based Panel Adhered by Different Adhesives

**Figure 2:**
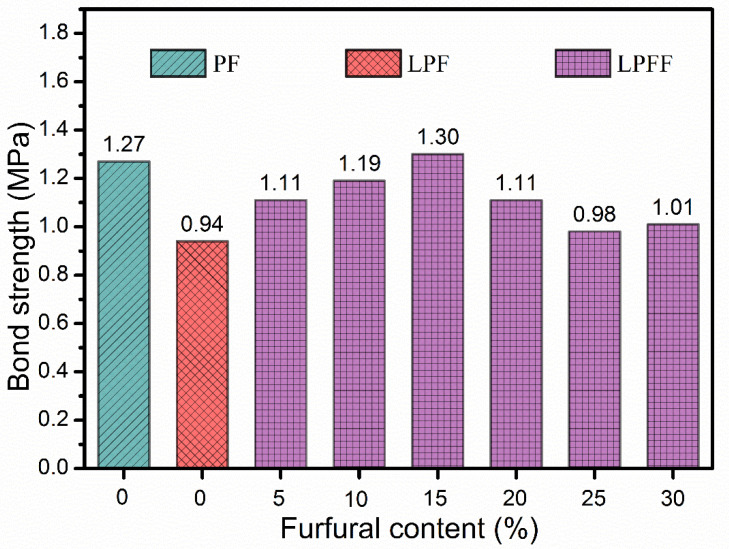
Effect of furfural content on shear strength.

Shear strength is an important parameter to determine the adhesion quality of wood-based panels [25], and it can be used to judge the water resistance of adhesives. The three-layer plywood specimen did not undergo delamination after two boiling water treatments, so a shear strength test was performed on a universal testing machine. Figure 2 shows the effect of furfural addition on the shear strength. The wood panels with different furfural contents exhibited a significantly higher shear strength than the LPF panel. The wet shear strength of LPFF15 (1.30 MPa) was 38.30% higher than that of LPF (0.94 MPa) and also better than that of PF adhesives. The results show the furfural addition was beneficial to improving the shear strength of the LPF adhesives, which may be related to the increase in crosslink density, and the formation of a complex network structure increased the water resistance [26]. Li et al. [27] used sodium sulfite as a catalyst to improve the demethylation efficiency of lignin, in order to improve the adhesive strength of lignin phenolic resin. As a result, the adhesive shear strength increased from 0.92 MPa to 1.07 MPa. Compared with the above report, the shear strength of the adhesive in this study reached 1.30 MPa, an increase of 21.50%. However, the shear strength of the LPF joint decreased when the furfural content was >15% (from 1.30 to 1.01 MPa). This outcome may be explained by the excess furfural not only was unable to effectively participate in the reaction, but also destroyed the stability of the original system. These results indicate that the appropriate furfural content was 15%.

### 3.2. Physicochemical Properties of Different Adhesives

**Figure 3:**
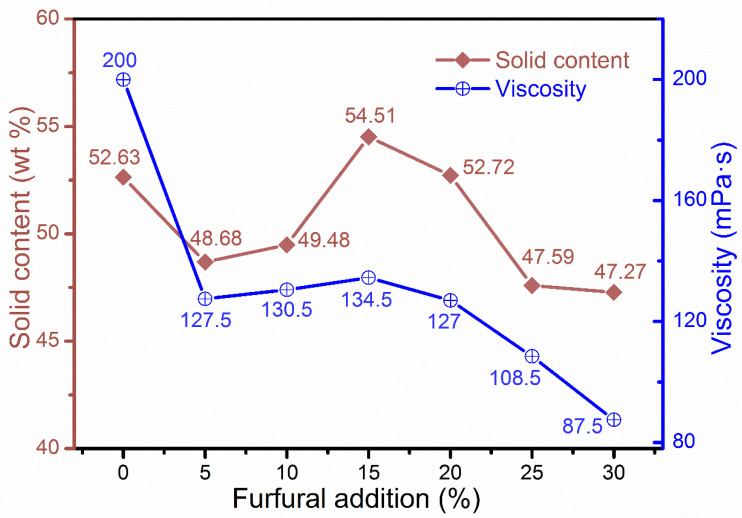
Effect of furfural content on solid content and viscosity.

The solid content, viscosity, free phenol, and free aldehyde contents of the adhesive determine its practical application value. Through the shear strength test, it was found that furfural was beneficial to improving the water resistance; therefore, the basic performances of LPF and LPFF adhesives were compared. The effect of furfural content on the solid content and viscosity is illustrated in Figure 3. Obviously, the molecular structure of furfural contains epoxy functional groups, which can be used as a diluent to efficiently reduce the LPF system viscosity [28]. When the furfural content was 5–15%, the viscosity (from 127.5 mPa·s to 134.5 mPa·s) and solid content (from 48.68% to 54.51%) showed an increased trend; as the furfural content further increased, both showed a downward trend. This is because the active site of furfural can react with the active site of phenolic compounds, so that more crosslinks are generated between condensation polymers, This results in more cross-linking between the polycondensates, which reduces the low molecular weight substances and thus increases the content of solidified products [29]. At the same time, the movement of the chain segment is hindered after crosslinking, which leads to the decrease of the flowability and the increase of the viscosity of the polymer. When the content of furfural is more than 15%, excessive furfural will affect the effective collision of molecules, which will reduce the rate of condensation reaction and eventually lead to the decrease of viscosity and solid content.

**Figure 4:**
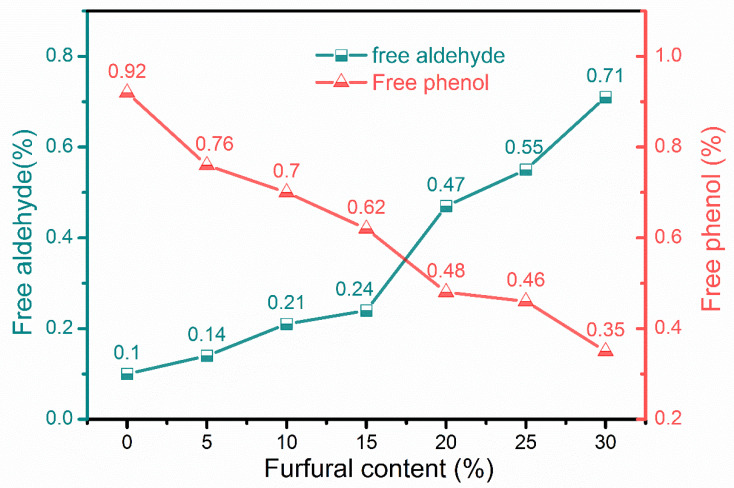
Effect of furfural content on free phenol and free aldehyde.

Figure 4 illustrates the effect of furfural content on free phenol and free aldehyde. There in, the free aldehyde content includes the free furfural content and the free formaldehyde content. The level of free aldehyde increased as a result of only part of furfural participated in the reaction. Moreover, with the increase in the furfural content, the free phenol content decreased from 0.92% to 0.35%. Under certain conditions, the free aldehyde and free phenol in the adhesive are a pair of contradictions, which cannot be increased at the same time [30]. The free aldehydes increased and the free phenols decreased gradually, which indicated this point.

Based on the above analysis, 15% furfural content as the crosslinking agent in the LPF system was the optimal choice. At this content, the bond strength of the prepared LPFF15 was 1.30 MPa, the free aldehyde content was 0.24%, the free phenol content was 0.62%, the solid content was 54.51%, and the viscosity was 124.5 mPa∙s. The above properties meet the Chinese National Standard National Standard (GB/T 14732-2017), indicating that the prepared LPFF resin has good application prospects in wood adhesive manufacturing.

### 3.3. Possible Interaction between Furfural and LPF Adhesive

**Figure 5:**
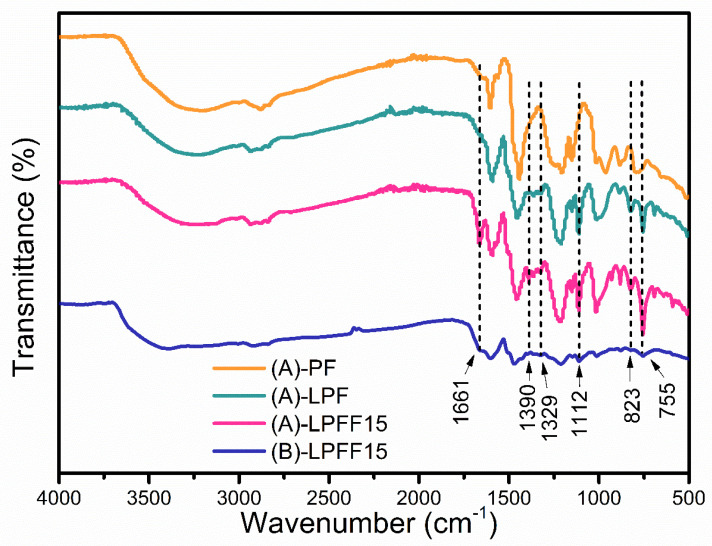
Infrared spectrograms of various adhesives; (A) means uncured and (B) means cured.

**Scheme 1:**
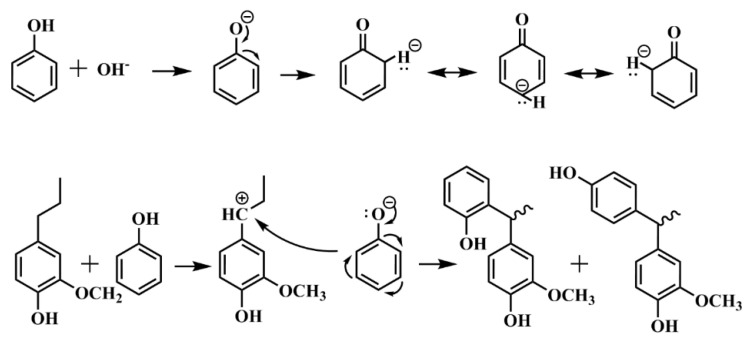
Phenolation of lignin and phenol under alkaline conditions.

The bonding performance of the resin depends on the resin chemical structure; thus, the crosslinking modification of furfural was verified by FTIR. The FTIR spectra of the PF, LPF, and LPFF15 are displayed in Figure 5, and Table 1 summarizes the assignments of each spectrum [31,32,33]. The absorptions at 1329 and 1112 cm^−1^ are attributed to lignin syringyl C-O stretching and syringyl aromatic ring C-H in-plane deformation, respectively [34]. The LPF spectrum has characteristic bands at 823 and 755 cm^−1^, attributed to the nucleophilic substitution reaction of phenol. As shown in Scheme 1, phenol ionizes out H^+^ into phenoxy anions under alkaline conditions. The transfer of electrons on the oxygen atom to the benzene ring makes the ortho-para position of the phenolic hydroxyl group negatively charged; this is due to the P-π conjugation between the lone pair of electrons of the oxygen atom and the large π bond of the benzene ring. Phenoxy anions act as nucleophiles to attack the central carbon atom of lignin, which significantly increases the active site of lignin. For the LPFF15, the intensity of the benzene ring at 755 cm^−1^ is significantly higher, indicating that the active site of furfural interacted with phenolic lignin. Compared with LPF and PF, LPFF15 displays new characteristic peaks of amide at 1661 and 1390 cm^−1^, due to the conjugated carbonyl C=O group of furfural and furfural double bonds, respectively. It could be found that the carbonyl peak intensity of the cured LPFF15 adhesive decreased significantly at 1661 cm^−1^. This should be caused by the condensation reaction of the carbonyl groups during the curing process of the adhesives. The FTIR result confirms that furfural was successfully introduced into the phenolic crosslinking system.

**Figure 6:**
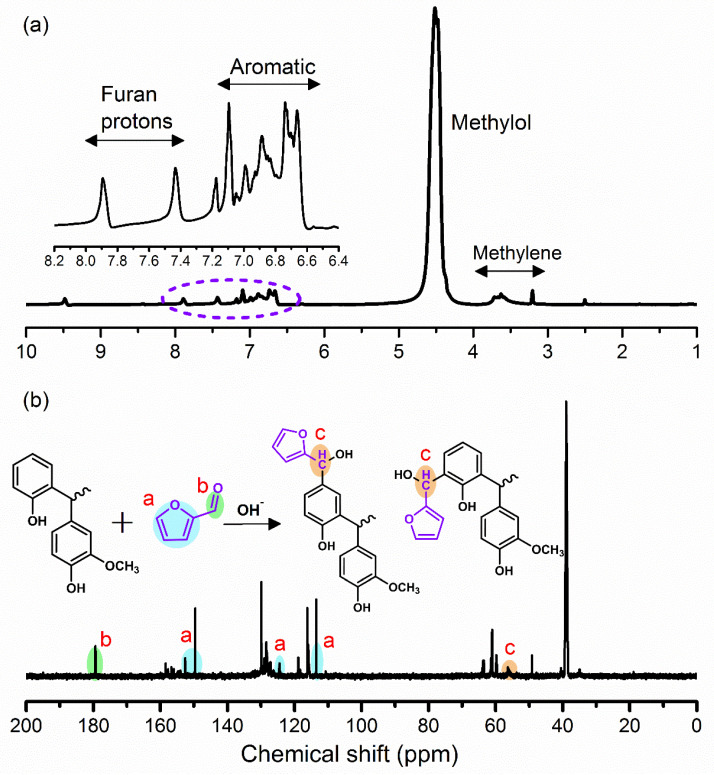
(**a**) ^1^H-NMR spectra of furfural modified lignin phenolic (LPFF) resin with 15% furfural content; (**b**) ^13^C-NMR spectra of furfural modified lignin phenolic (LPFF) resin with 15% furfural content.

To further reveal the chemical reaction mechanism of the crosslinked polymer, the polymer was evaluated via ^1^H-NMR and ^13^C-NMR techniques. The ^1^H-NMR spectrum of the LPFF15 adhesive is displayed in Figure 6a. The intense signal at 7.1–6.7 ppm was due to the hydrogen on the benzene ring. The proton signals were 9.5 ppm (aldehyde hydrogen), 7.9 ppm, and 7.4 ppm (furan hydrogen), related to the corresponding protons of the furfural [35]. The characteristic peaks corresponding to saturated hydrogen at 4.5–3.2 ppm (CH_2_, CH_2_OH, CH) provide support for the nucleophilic addition reaction of furfural with phenol ring.

**Scheme 2:**
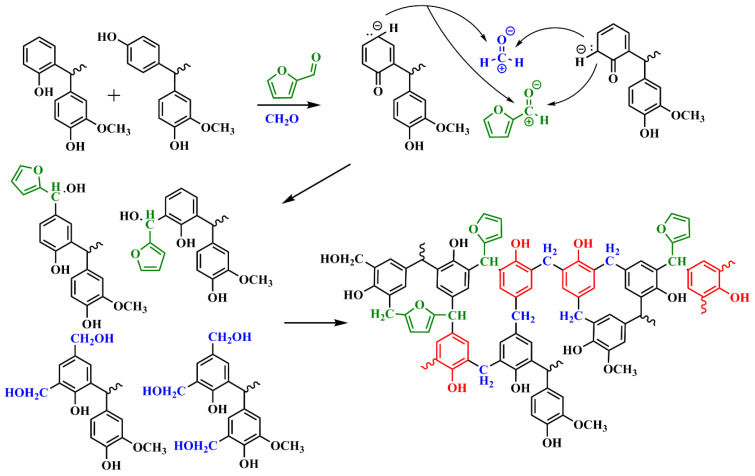
Synthesis mechanism of LPFF adhesive.

Figure 6b shows the ^13^C-NMR spectra of the LPFF15 adhesive. The signal peak between 154 ppm and 158 ppm corresponds to the ortho-para position with the substituent phenolic hydroxyl group, while the signals between 115 and 130 ppm may correspond to the ortho-para position with or without the substituent lignin phenol ring. A methylene bridge was observed at 40.7–34.9 ppm. Moreover, several significant peak changes caused by furfural were observed. The characteristic peaks at 179.4, 152.6, and 113.5 ppm were due to the aldehyde-based carbon of furfural, the ortho-position of furfural, and the carbon of the furan ring, respectively. These findings suggest that part of the unbound furfural can be further crosslinked and cured under heating conditions. The signals at 149.6 ppm (methylene bridge) and 56 ppm (methine bridge) correspond to the polycondensation between the ortho-position of furfural and the phenol ring [7,36]. Phenolic lignin was connected to furfural with –CHF–OH (F is for furan ring) as the crosslinking structure, and various hydroxymethylphenols and formaldehyde were formed. After the macromolecular substance further crosslinked with –CH_2_- and –CH–, a network structure was formed (Scheme 2). In conclusion, it was confirmed that the resin reaction between furfural and the lignin-phenol ring increased the crosslinking density and effectively inhibited the molecular structure damage by water.

### 3.4. Thermal Stability Analysis

**Figure 7:**
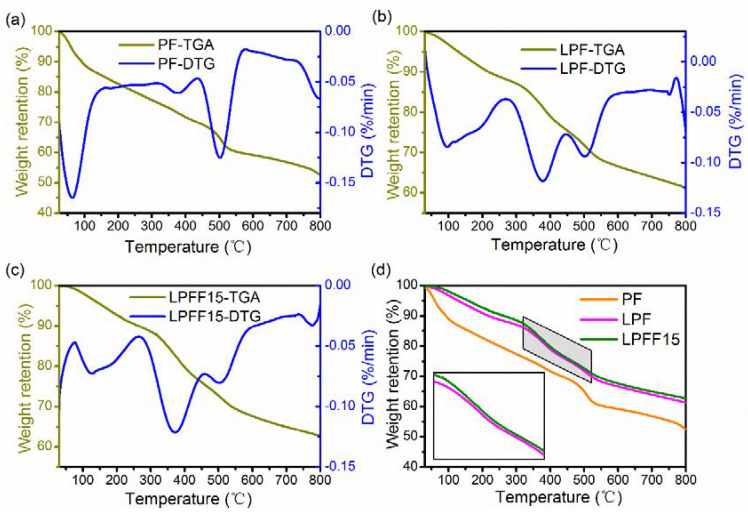
(**a**) Thermogravimetric analysis (TGA) and derivative thermogravimetry (DTG) of phenolic formaldehyde (PF) resin adhesive; (**b**) TGA and DTG of lignin-phenol-formaldehyde (LPF) resin adhesive; (**c**) TGA and DTG of LPFF resin adhesive with 15% furfural content; (**d**) thermogravimetric curves of various adhesives.

The TGA and DTG curves of the PF, LPF, and LPFF15 adhesives are displayed in Figure 7. The results indicate that the thermal degradations of LPFF15 and LPF adhesives were similar to that of the PF adhesive under the temperature range of 30–800 °C. In the initial stage, the weight loss at temperatures below 265 °C observed for the adhesive can be ascribed to the evaporation of water; it was also caused by the volatilization of small molecular substances such as free aldehyde and free phenol in the resin [37]. Owing to the relatively large molecular weight and stable furan ring structure of furfural, LPFF15 had a better thermal stability. The main weight loss occurred at about 265 °C to 550 °C. This was caused by the breakage of the methylene and methine bridges in the molecular chain. Moreover, lignin would degrade at a faster rate within this temperature range [38]. After 550 °C, the weight loss was due to the further degradation of the polymer and the volatilization of small molecules, such as carbon monoxide and carbon dioxide [39]. In the final stage, the carbon yields of LPF and LPFF15 at 800 °C were 61.3% and 62.6%, respectively. Furthermore, Carlos et al. [40] synthesized *Jatropha curcas* seed-husk lignin phenolic resin, and its carbon residue rate reached 60% at 514 °C. Thus, furfural has a better effect on the thermal stability of lignin phenolic resin. These differences occurred because the furfural and the phenolic compound formed a larger and more complex crosslinked network structure, enabling the superior thermal stability of the adhesive.

### 3.5. Analysis of Plywood Shear Surface Morphology

**Figure 8:**
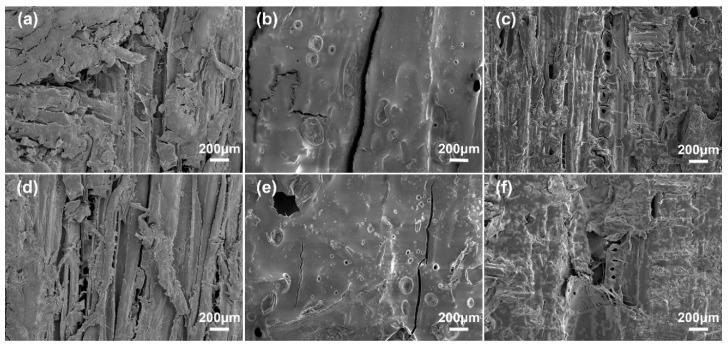
Micrographs of bonded joints after the bond strength test. (**a**,**d**) are PF-bonded joint morphologies, (**b**,**e**) are LPF-bonded joint morphologies, and (**c**,**f**) are LPFF-bonded joint morphologies with 15% furfural content.

The micrographs of bonded joints after the wet shear strength test (Figure 8) revealed differences in the mechanical properties of the LPFF15, LPF, and PF adhesives. As shown in Figure 8, no wood fiber was observed on the LPF-bonded joint section, and there were many cracks caused by the fracture of the glue layer, indicating that the adhesive did not penetrate into the wood interior. Brittle fracture of the glue layer occurred under the action of external force, and a small shear strength was obtained. In addition, the glue section had numerous unevenly distributed holes; the water resistance was weakened owing to the water infiltration through the holes in the adhesive layer surface, which is similar to the finding of a previous report [41]. The morphologies of PF-bonded joints (Figure 8a,d) and LPFF15-bonded joint (Figure 8c,f) were significantly different from that of the LPF-bonded joint (Figure 8b,e). As observed from the glued section of the LPFF15 and PF joints, it was found that there were many tearing bearing surfaces in wood, and the adhesive adhered to the wood catheter and wood rays. This indicates that the adhesive modified by furfural gradually infiltrates into the interior of wood to the formation of glue nails, which improve the shear strength of plywood. Therefore, the mechanical characteristics of LPFF were confirmed by the morphology of its glued section, indicating that adding furfural to LPF adhesive was beneficial to the adhesion between glue and wood. This result is consistent with the rise in shear strength in the water resistance test.

## 4. Conclusions

With furfural as a crosslinking agent, high-biomass-content water-resistant lignin-based wood adhesive was successfully prepared under alkaline conditions. Physical and mechanical property tests and SEM image analysis indicated that the LPFF adhesive with 15% furfural content had better shear strength and water resistance than LPF. Through FTIR, ^1^H-NMR, and ^13^C-NMR analyses, the possible mechanism of polymerization reaction formation of the LPFF adhesive was inferred. The formation of -CHF-OH (F is for furan ring) as the crosslinking structure was found to be due to a nucleophilic addition reaction between the ortho-position of the phenolic hydroxyl group in phenolic lignin and furfural. Thermogravimetric analysis revealed that the LPFF adhesive had good thermal stability. The significant improvement in the thermal stability of LPF adhesives is attributed to the formation of larger and more complex crosslink network structures of furfural and phenolic compounds. In addition, in terms of physical properties, the incorporation of furfural led to an increase in the solid content of lignin phenolic resin (from 0 to 15%) and a decrease in viscosity. Given the above, LPFF adhesives have broad application prospects in the area of wood adhesives, which will facilitate the efficient use of biomass resources.

## Figures and Tables

**Table 1:** Assignments of Fourier-transform infrared spectra of various adhesives.

Assignments	(A)-PF	(A)-LPF	(A)-LPFF15	(B)-LPFF15
	Wavenumber (cm^−1^)			
–OH groups stretching	3203	3216	3234	3387
C–H	2880	2934	2934	2927
Carbonyl C=O group of furfural	-	-	1661	1661
Aromatic ring structure stretching	1605	1594	1594	1604
–CH_2_ bridge	1443	1455	1455	1467
Furfural double bond	-	-	1391	-
Syringyl C–O stretching	-	1326	1328	1325
Conjugate C–O groups stretching	1206	1212	1212	1212
Syringyl aromatic ring C–H in-plane deformation	-	1112	1112	1112
Unconjugated C–O groups stretching	1014	1013	1016	1012
C–H out of plane, para-substituted	-	823	824	823
C–H out of plane, ortho-substituted	-	755	755	755
